# Vascular and Neuronal Network Formation Regulated by Growth Factors and Guidance Cues

**DOI:** 10.3390/life13020283

**Published:** 2023-01-19

**Authors:** Yuki Wakayama, Satoru Yamagishi

**Affiliations:** 1 Max Planck Institute for Molecular Biomedicine, Roentgenstrasse 20, 48149 Muenster, Germany; 2 Department of Biology, Friedrich Alexander Universität Erlangen-Nürnberg, Strasse 5, 91058 Erlangen, Germany; 3 Department of Organ and Tissue Anatomy, Hamamatsu University School of Medicine, Hamamatsu 431-3192, Japan; 4 Optical Neuroanatomy, Preeminent Medical Photonics Education & Research Center, Hamamatsu University School of Medicine, Hamamatsu 431-3192, Japan

**Keywords:** blood vessel formation, neuronal formation, guidance molecules

## Abstract

Blood vessels and nerves are distributed throughout the body and show a high degree of anatomical parallelism and functional crosstalk. These networks transport oxygen, nutrients, and information to maintain homeostasis. Thus, disruption of network formation can cause diseases. Nervous system development requires the navigation of the axons of neurons to their correct destination. Blood vessel formation occurs via vasculogenesis and angiogenesis. Vasculogenesis is the process of de novo blood vessel formation, and angiogenesis is the process whereby endothelial cells sprout from pre-existing vessels. Both developmental processes require guidance molecules to establish precise branching patterns of these systems in the vertebrate body. These network formations are regulated by growth factors, such as vascular endothelial growth factor; and guidance cues, such as ephrin, netrin, semaphorin, and slit. Neuronal and vascular structures extend lamellipodia and filopodia, which sense guidance cues that are mediated by the Rho family and actin cytosol rearrangement, to migrate to the goal during development. Furthermore, endothelial cells regulate neuronal development and vice versa. In this review, we describe the guidance molecules that regulate neuronal and vascular network formation.

## 1. Introduction

Each organ consists of numerous cell types and various structures; thus, their development is regulated temporally and spatially. The central nervous system (CNS) is part of the nervous system and consists of the brain and the spinal cord. Because the functional cerebral cortex has a complex structure, its development is coordinated in an elaborated manner. During development, commissural axons, which connect the left and right hemispheres of the brain, allow communication between both sides of the body. Regulatory mechanisms that enable these commissural axons to determine and follow appropriate trajectories from one side to the other are important. Brain development requires neurons to migrate and position themselves correctly, which demands progenitor cells to migrate from the site of origin in the early neural tube to the final site of differentiation in multilayered brain tissues. A typical neuron has a long axon and branched dendrites that enable them to create extensive synaptic connections to other neurons in various regions. We describe the guidance molecules involved in these axonal projections and cortical migrations.

The vertebrate retina has multiple layers: the inner limiting membrane, optic nerve fiber layer, ganglion cell layer, inner plexiform layer, inner nuclear layer, outer plexiform layer, outer nuclear layer, external limiting membrane, photoreceptor layer, and retinal pigment epithelium. Astrocytes in the retina generate vascular endothelial growth factor (VEGF) to promote angiogenesis. The inner retinal vascular plexus develops in close association with the pre-existing layer of astrocytes. Astrocyte scaffolds play a major role to guide the endothelial filopodia extension in retinal vessel pattern formation. Age-related macular degeneration is an acquired dysfunction of the macula, characterized by progressive visual impairment. Ectopic expression of VEGF promotes the outgrowth of abnormal vessels underneath the photoreceptor layer and under the retinal pigment epithelium. Anti-VEGF antibody treatment is one candidate therapy [1].

Physiological development is controlled by specific inductive signals and transcription regulators. However, pathological events, such as tumor angiogenesis, are not programmed genetically. For instance, tumor cells secrete growth factors to solve hypoxia. In contrast to physiological development, endothelial barrier function is not organized because cell adhesion is weak and vessels are not covered with mural cells. Therefore, the vasculature formed following pathological events is leaky. We discuss the signaling cascades of VEGF and other axonal guidance molecules that regulate angiogenesis and neuronal guidance.

## 2. VEGF

In 1971, Judah Folkman proposed that the growth of solid neoplasms is accompanied by neovascularization and tumor angiogenesis is the potential therapeutic benefit [2]. In 1989, VEGF was isolated and identified as endothelial mitogen [3].

Growth factors, including VEGF, and guidance cues regulate the actin cytoskeleton via Rho guanosine triphosphatases (GTPase) to modulate cell polarity and motion. Downstream Rho GTPases are activated by guanine nucleotide exchange factors (GEF) and inactivated by GTPase-activated proteins (GAP).

VEGF is a secreted homodimeric glycoprotein. VEGF/VEGF receptor (VEGFR) signaling plays important roles in angiogenesis and neurogenesis. The VEGF family includes VEGF-A, VEGF-B, VEGF-C, VEGF-D, VEGF-E, and placental growth factor (PlGF) [4]. VEGF-A comprises several subtypes in humans: 121, 165, 189, and 206 amino acid peptides. VEGF-A_165_ and VEGF-A_189_ have an affinity for heparin sulfate, and VEGF-A_165_ is the most common VEGF-A. The interaction between VEGF-A and the extracellular matrix restricts their distributions [5].

Hypoxia induces the expression of VEGF-A to deal with ischemia. Hypoxia-inducible factors (HIFs) are depredated rapidly by protease under normoxia conditions and stabilized under hypoxia. The VEGF-A promoter region has a hypoxia-response element (HRE) that binds to HIFs [4,6]. VEGF/VEGFR signaling affects not only physiological angiogenesis but also pathological angiogenesis. However, VEGF-A_165b_, a VEGF-A splicing variant, has been shown to inhibit the revascularization of ischemic hind limbs in mice. This isoform is derived from differential splicing of exon8 [7].

VEGF-A binds to VEGFR1 and VEGFR2, whereas PlGF and VEGF-B bind only to VEGFR1. VEGF-C and VEGF-D can bind to VEGFR2, although their affinity for VEGFR2 is lower than that for VEGFR3 [8,9]. Endothelial cells also secrete VEGF-A to maintain vessel homeostasis. Furthermore, in mice, endothelial cell-specific VEGF-A knockdown causes progressive endothelial degeneration and sudden death by 25 weeks of age [10].

VEGF-C and VEGF-D play major roles in lymphangiogenesis. To enable the full function of VEGF-C, VEGF-C is cleaved by A disintegrin and metalloprotease with thrombospondin motifs 3, collagen, and the calcium-binding epidermal growth factor domains 1 (CCBE1) complex [11]. In zebrafish, lymphatic formation starts 32 h postfertilization. Lymphatic precursor cells sprout from the cardinal vein and migrate dorsally. CCBE1 knockout zebrafish, which are phenocopies of VEGF-C and VEGFR3 knockout fish, exhibit a complete loss of lymphatic vessels [12,13].

**Figure 1:**
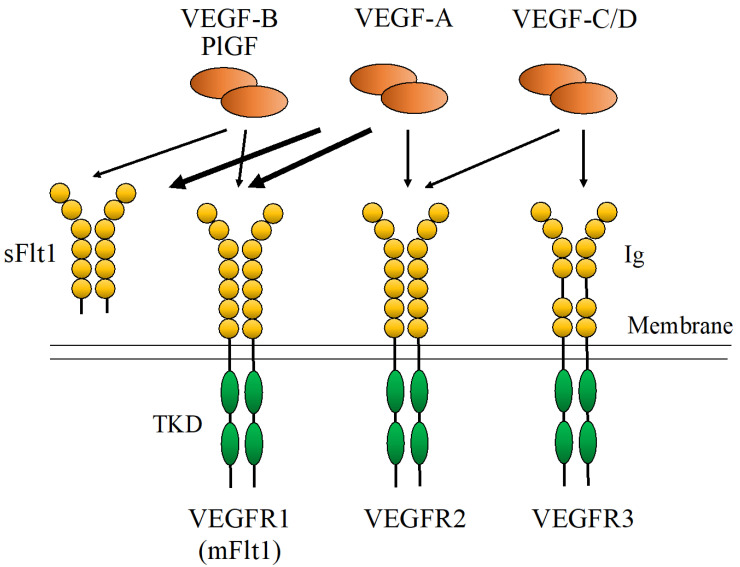
Structure of the vascular endothelial growth factor (VEGF)/VEGF receptor (VEGFR), their interaction, and their binding pattern. TKD: tyrosine kinase domain.

The VEGFR family has three members: VEGFR1 consists of an extracellular domain harboring immunoglobulin-like domains, a transmembrane domain, and a tyrosine kinase domain (Figure 1), and VEGFR2 and VEGFR3 comprise similar domains. VEGFR2 is a major signal transducer that promotes angiogenesis. In contrast to VEGFR2, VEGFR1 binds to VEGF-A with high affinity; however, the tyrosine kinase activity of VEGFR1 is approximately 10-fold weaker than that of VEGFR2. Therefore, VEGFR1 plays a negative role in angiogenesis [8]. Furthermore, VEGFR1 has two isoforms: soluble fms-like tyrosine kinase 1 (sFlt1) and membrane-bound fms-like tyrosine kinase 1. SFlt1 is soluble and lacks a tyrosine kinase domain, and thus functions as a VEGF-A decoy [4].

VEGFR1 knockout mice show overgrowth of endothelial cells and dysfunction of blood vessels, which sequentially causes embryonic lethality [8]. VEGF-A/VEGFR2 signaling promotes endothelial migration, differentiation, and proliferation. VEGFR2 Y1175 is a major phosphorylation site, where after autophosphorylation, the receptor binds to Phospholipase C-γ (PLC-γ) and activates the Raf-MEK-MAP kinase pathway. Phosphorylation of Y1175 is required for Phosphoinositid-3-Kinase (PI3K)/Akt activation. Akt promotes the survival of endothelial cells and nitric oxide (NO) production with the help of endothelial NO synthase [4,14].

The spinal cord neurons of zebrafish secrete Vegfaa and sFlt1 to restrict Vegfaa-kinase insert domain receptor like (kdrl) signaling. Spinal cord vascularization occurs from the veins involving two-tiered regulation of neuronal sFlt1 and Vegfaa via a novel sprouting model [15].

During eye development, VEGFR2 is more abundantly expressed in retinal neurons than in endothelial cells [16]. Notably, the genetic deletion of VEGFR2 in neurons leads to angiogenesis that is misdirected toward neurons and an increase in vascular density around neurons, which indicates crosstalk between neurons and vessels. However, VEGFR2 knockdown in neurons does not affect neuronal development. Neuron-derived VEGF likely contributes to cortical and hippocampal development in an angiogenesis-independent manner and via a direct neurotrophic effect mediated by VEGFR1 and VEGFR2 in neurons [16].

The concentration of VEGF-A is a crucial factor because angiogenesis is regulated by the fine tuning of VEGF-A concentration. In the mouse retina, secreted VEGF-A accumulates along the astrocyte track, and the localization of VEGF-A is regulated by the heparin-binding domain of VEGF-A. Ectopic overexpression of VEGF-A induces abnormal filopodia guidance in tip cells. In addition, the ectopic VEGFR2 agonist induces stalk cell proliferation and inhibits peripheral growth of the retina. These findings suggest that the spatial distribution of secreted VEGF-A is critical for maintaining a balance between capillary branching and vessel growth [17,18]. VEGF-A homozygote and heterozygote knockout mice are lethal. Moreover, the overexpression of VEGF-A is also lethal.

During retinal development, the spreading of the inner vascular plexus originates from the optic disk and extends to the peripheral margin. The vascular branches extend from the inner plexus to the retina to form the outer plexus.

VEGFR3 is highly expressed in lymphatic endothelial cells. VEGF-C and VEGFR3 are crucial for lymphangiogenesis. VEGF-C promotes lymphatic endothelial cell differentiation, expresses Prox1 from veins, and proliferates lymphatic endothelial cells. VEGF-D is also involved in lymphangiogenesis; however, it is not essential for lymphatic development [19].

In zebrafish, VEGF signaling regulates artery venous segmentation, whereby venous angioblasts sprout ventrally from the dorsal aorta. VEGF-A activates Delta-like protein (dll4)-Notch signaling and subsequently promotes arterial differentiation in a subset of angioblasts. VEGF-A knockdown by morpholino (MO) inhibits artery specification. Furthermore, PLC-γ morphants, downstream of VEGF-A signaling, show a similar phenotype [20].

Healthy vessels show organized hierarchical patterning and tight cell adhesion supported by basal membrane and mural cells. In contrast, tumor vessels show an unorganized network due to the imbalanced expression of angiogenic factors. In addition, weak cell junctions and the loss of mural cell coverage cause vessel leakage [21]. Knowledge regarding VEGF signaling can reveal valuable regulatory mechanisms underlying physiological and pathological angiogenesis. In clinical research, anti-VEGFR2 antibodies and VEGFR2 inhibitors have contributed to therapies for tumors and age-related macular degeneration [22,23]. Below, we discuss other guidance molecules that regulate vascular and neuronal formation.

## 3. Ephrin/Eph

Guidance molecules play attractive and/or repulsive roles during neuronal and vascular development. Ephrin has two subtypes: ephrinA1–ephrinA5, which are tethered to the cell membrane by glycosylphosphatidylinositol (GPI), and ephrinB1–ephrinB3, which have a transmembrane domain. EphA receptors bind to most ephrinAs, and EphB receptors bind to most ephrinBs, with the exception of the EphA4 receptor, which can bind to both ephrinAs and ephrinBs [24].

**Figure 2:**
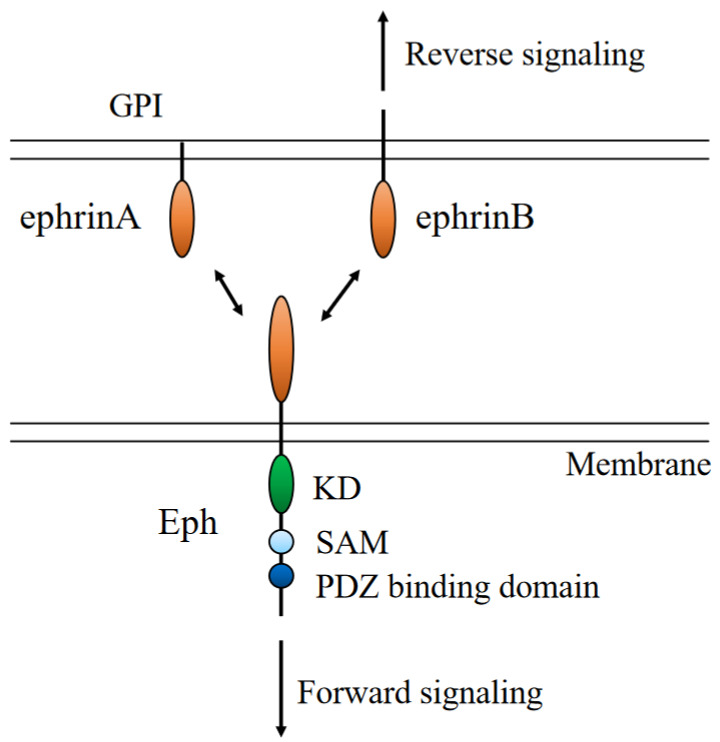
Structure of ephrin/Eph, their interaction, and their domain structure. GPI: glycosylphosphatidylinositol; LBD: ligand-binding domain; RBG: receptor binding domain; EGF: epidermal growth factor; TK: tyrosine kinase; SAM: sterile alpha motif.

The Eph receptor is a receptor tyrosine kinase (RTK). Eph receptors consist of an extracellular globular ligand-binding domain, a Cys domain, fibronectin domains, a transmembrane domain, an intracellular tyrosine kinase domain adjacent to a sterile alpha motif, and a postsynaptic density-95, discs-large, zona occludens 1 (PDZ) domain (Figure 2).

Forward signaling is induced by a ligand binding to RTKs. Because ephrin ligands are membrane-bound, they act as receptors, which is termed reverse signaling [25].

The cytoplasmic domain of ephrinB comprises five conserved tyrosine residues. These tyrosines are phosphorylated by the Src family (i.e., Src and Fyn) upon EphB binding. This phosphorylation is important for reverse signaling. Adapter proteins, such as Growth factor receptor-bound protein 4 (Grb4), bind to phosphorylated ephrinB and subsequently induce rearrangement of actin cytoskeleton focal adhesion. Ephrin/Eph form clusters where there is cell–cell contact to enhance signaling. EphrinB2 and EphB4 are highly expressed in the artery and vein, respectively, and the removal of ephrinB2 or EphB4 results in various cardiovascular defects [26]. In zebrafish, ephrinB2a, an ortholog of ephrinB2, is highly expressed in the dorsal cells of the vascular cord. EphrinB2a MO and Ephb4 MO disturb segregation from the common vessel primordium [20].

Ephrins not only work as attractive/repulsive signals but also regulate receptor endocytosis. In the retina, ephrinB2 regulates the turnover of VEGFR2 via Disabled-2 (Dab2) and partitioning defective 3 (PAR3) [27]. Furthermore, ephrinB2 induces VEGFR3 internalization to regulate angiogenesis and lymphoangiogenesis [28].

In the visual system, topographic mapping of retinal axons along the anterior–posterior axis depends on repulsion mediated by ephrinA ligands and Ephs. EphrinA is expressed along a gradient in the tectum, and EphA is expressed along a complementary gradient in the retina [29]. EphrinA5 knockout mice exhibit defects in neural tube closure, and EphrinB3 works as a cell-attached midline repellent for EphA4-expressing neurons (e.g., corticospinal tract neurons) [30].

Ephrin/Eph signaling is coupled with the Rho family. Small GTPase Rac activity is required for ephrin-/Eph-induced membrane ruffling and cell repulsion, and Small GTP ase Cdc42 is required for filopodia formation and dendritic spine formation.

Ephexin is a GEF that binds to EphA4. The EphA4 kinase domain interacts with the Dbl homology–pleckstrin homology region of Ephexin. Inhibition of Ephexin disturbs ephrinA-induced growth cone collapse. Moreover, Ephexin activates small GTPase RhoA but inhibits Cdc42 and Rac1 [29,31,32].

Other GEFs are also regulated by Eph signaling. Guanine nucleotide exchange factor Vav2 can bind to the intracellular domain of EphA4 and EphB2 via its Src homology 2 (SH2) domain and phosphorylates tyrosine in response to ligand binding. In addition, Vav2 and Vav3 double-knockout mice show axon guidance defects [24,33].

## 4. Netrin/Uncoordinated 5 (UNC5)/Fibronectin Leucine-Rich Transmembrane Protein (FLRT)

**Figure 3:**
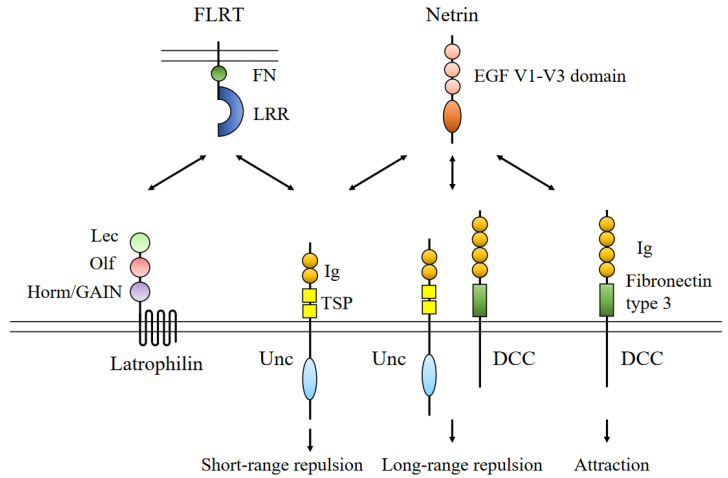
Structure of netrin/the receptors and their interaction, and the domain structures of netrin, deleted in colorectal cancer (DCC), and Uncoordinated 5 (UNC5). Ig: immunoglobulin; TPS: thrombospondin-1; Lec: lectin; Olf: olfactomedin-like; LRR: leucine-rich repeat.

Netrin is a prototypical axon guidance molecule. The netrin family consists of Netrin-1, -3, -4, and -5, and GPI-anchored netrin-G1 and -G2 in mammals. These molecules bind to UNC5A-D, deleted in colorectal cancer (DCC), neogenin, and netrin-G ligands. Netrin produces attractive and repulsive signals depending on the receptors involved and consists of three epidermal growth factor-like repeat domains and laminin V and VI domains (Figure 3).

Netrin-1 is secreted from the floor plate and ventricular zone (VZ) of the spinal cord. It diffuses through the extracellular matrix and establishes a gradient that attracts growing commissural axons toward the ventral midline of the spinal cord. In mammals, there are six netrin-1 receptors. Aside from UNC5A-D, DCC and neogenin are members of the immunoglobulin superfamily, which carry extracellular fibronectin type III repeats.

DCC is a transmembrane protein that comprises P1, P2, and P3 domains. The P1 domain binds with the DCC binding (DB) domain of UNC5. Netrin consists of a laminin VI domain, laminin V-type EGF-like domains, and a netrin (NTR)/C345C domain, except for netrin-5, which lacks a laminin VI domain.

Netrin has two functions: attracting and repelling axons during CNS development. When netrin-1 binds to DCC, it functions as an attractant signal, whereas when it binds to UNC5, it functions as a repulsive signal in axonal outgrowth. Furthermore, netrin-1 binding to DCC and UNC5 functions as a long-range repulsive signal. Netrin-1 expressed in the VZ is required for the growth of commissural axons and functions as a local cue but not as a long-range attractant, as was initially believed [34]. Moreover, netrin-1 promotes angiogenesis mediated by RhoA. The binding of netrin-1 to UNC5B inhibits angiogenesis. Netrin-1a regulates the copatterning of vessels and nerves as it navigates motoneuron axons, which is crucial for normal vessel sprouting in fish. Furthermore, netrin-1 and netrin-4 promote tube formation, proliferation, and migration in cultured endothelial cells, and increase the capillary density of the vasa nervorum [35].

UNC5B is highly expressed in developing blood vessels, especially in arteries, capillaries, and tip cells. UNC5B knockout mice show an increase in the number of vessel branches and filopodia in the CNS [36]. Furthermore, an injection of recombinant netrin-1 induces the retraction of filopodia in tip cells and reduces the number of tip cells. In zebrafish, knockdown of UNC5b or netrin-1a increases the number of branches in segmental vessels (ISV) [36].

The FLRT family is a type I transmembrane protein and consists of an extracellular leucine-rich repeat (LRR) sequence, type III fibronectin domains, and cytoplasmic tails with small GTPase Rnd1 binding motifs. FLRT works as not only a cell adhesion protein but also a repulsive axon guidance molecule. The N-terminus of FLRT is cleaved by metal protease, and the N-terminus of FLRT2 binds to UNC5D, whereas that of FLRT3 binds to UNC5B during neuronal guidance [37]. The above binding functions as a repulsive signal. FLRT2 knockout mice show defects in controlled neuronal migration in the brain. Vascular endothelial cell-specific conditional FRLT2 knockout mice have embryonic lethality in the B6 background during the midgestation stage with systemic congestion and hypoxia. Furthermore, FLRT2 mediates tumor-specific interendothelial adhesion via homophilic binding, which enhances cancer aggressiveness [38]. FLRT3 knockout mice are lethal because of dysfunctions in several aspects of embryonic development [39] In addition, FLRT1/3 double-knockout mice exhibit ectopic cortical gyrus formation. FLRT has an N-terminal extracellular LRR domain and a fibronectin-like III domain. The LRR domain interacts with latrophilin (Lphn) and G-protein-coupled receptors (GPCR). Lphn has an N-terminal lectin domain, an olfactomedin-like domain, and a Hornm/GPCR autoproteolysis-inducing domain. Lphn1 and Lphn3 are highly expressed in the CNS, and an Lphn3 mutation in humans is associated with attention-deficit hyperactivity disorder [40,41].

## 5. Slit/Robo

**Figure 4:**
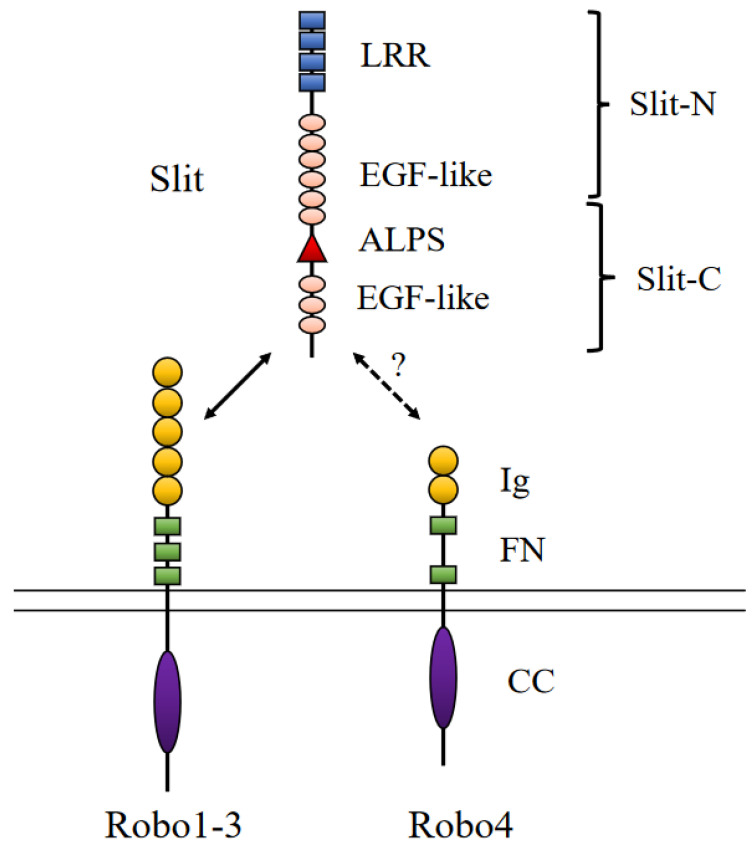
Structure of the Slit/Robo and their interaction. The epidermal growth factor (EGF)-like domain of Slit is cleaved by a protease. Slits bind to Robo1–3. However, it remains unclear whether Slits bind to Robo4. Slit-N: N-terminal domain of Slit; Slit-C: C-terminal domain of Slit; LRR: leucine-rich domains; EGF: epidermal growth factor; Ig: immunoglobulin; FN: fibronectin; CC: conserved cytoplasmic domain; LG: laminin G.

Slit is a secreted glycoprotein, and Robo is the receptor. Slit consists of an N-terminal signal peptide, leucine-rich domains, six epidermal growth factor-like domains, epidermal growth factor domains, and a C-terminal cysteine-rich knot. It is approximately 1500 amino acids long and is cleaved by an enzyme to generate a long N-terminal Slit segment and a short C-terminal Slit segment. The N-terminal ectodomain binds to Robo, whereas the C-terminal binds to the plexin receptor to regulate neuronal axonal migration. Slit binds to Robo via the immunoglobulin (Ig) 1 and Ig2 domains. Robos are transmembrane proteins and do not possess an autocatalytic domain. The extracellular domain of Robo1, 2, and 3 comprises five immunoglobulin-like domains and three fibronectin repeats [42] (Figure 4). Both Slits and Robos can bind to heparin sulfate proteoglycans. Slit-Robo elicits the repulsion of axons during neuronal development; however, in endothelial cells, this pathway inhibits or promotes angiogenesis depending on the cellular context. In the spinal cord, Slit is expressed in ventral midline cells, and commissural axons are first attracted and repelled by Slit after they cross the midline by switching isoforms from Robo3.1 to Robo3.2 [43].

Robo1 knockout mice have longer neurites in the subplate (SP) and subventricular zone (SVZ) [44]. Slit1 and Slit2 double-knockout mice exhibit defects in midline guidance in the spinal cord. Slit1 and Slit2 act as negative regulators of neuronal outgrowth in migrating interneurons [45] Robo4 is an endothelial cell-specific receptor that controls angiogenesis and stabilizes vasculature during pathological angiogenesis. Knockdown of Robo4 by small interfering RNA (siRNA) reduces IL-6 expression in endothelial cells [46]. Robo4 interacts with either Slit2 or UNC5B to maintain vascular integrity. Endothelial cells also express Robo1/2 and Slits to regulate cell migration and polarity. Furthermore, smooth vascular muscle cells and endothelial cells secrete Slit2 and Slit3.

Endothelial cells guide neuronal migration in the adult brain. New neurons generated in the SVZ migrate to the olfactory bulb along the rostral migratory stream. After brain injury, neuroblasts in the VZ-SVZ migrate toward the lesion. However, regeneration ability is limited by the reactive astrocytes that express Robo2. Overexpression of Slit1 enhances neuronal migration during brain regeneration [47,48,49].

Two-hybrid screening has revealed that the Slit-Robo-GTPase activating protein (srGAP) interacts with the intracellular domain (C3 domain) of Robo. In mammals, there are four srGAPs: srGAP1, srGAP2, srGAP3, and Arhgap4. SrGAP1 interacts with RhoA and Cdc42 [44,50], whereas srGAP2 regulates Rac1 activity in the protrusions where two cells spatially overlap, which induces contact inhibition of locomotion. SrGAP3 interacts with Rac1 and Cdc42 [44,51]. In addition, Robo1 binds to Myo9bm RhoA-GAP, which inhibits lung cancer cell migration and invasion [52].

A previous study has shown that PlexinD1 binds to Slit2 during commissural axon guidance. FLRT3 binds to Robo1 to modulate the responsiveness of axons to Netrin [53]. After brain injury, neural stem cell-derived neuronal precursors in the VZ-SVZ migrate toward the lesion. Neurons use Slit1-Robo2 to approach a lesion during regeneration. Overexpression of Slit1 in neuroblasts promotes this migration toward the lesion and efficiently regenerates the neuronal circuit [47].

## 6. Semaphorin/Plexin

**Figure 5:**
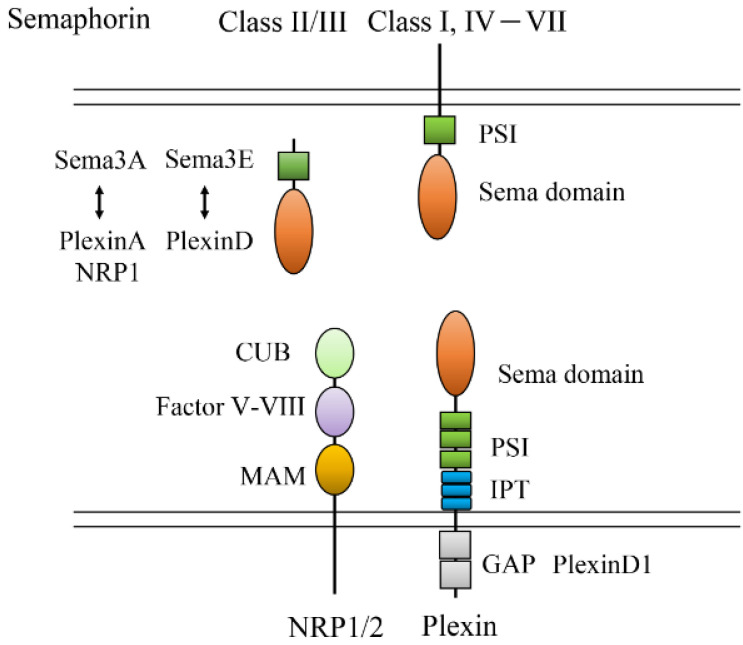
Structure of sema/plexin and their interaction. PSI: plexin, semaphorin, and integrin; CUB: complement C1r/C1s, Uegf, bone morphogenetic protein (Bmp); IPT: immunoglobulin domain shared by plexins and transcription factors; GAP: GTPase activating protein; TSP: Thrombospondin; CUB: C1r/C1s, uEGF, bone morphogenetic protein; MAM: meprin, A5, and Receptor protein-tyrosine phosphatase μ; PSI: plexin-semaphorin-integrin.

Semaphorins were originally identified as axonal growth cone guidance molecules and have eight subclasses: classes I and IV–VII are membrane-associated and classes II, III, and VIII are secreted. Plexin is a receptor for semaphorin and a transmembrane protein that encodes the R-Ras GAP domain (Figure 5) [54]. Most membrane-bound semaphorins bind to plexins directly, although class III semaphorins bind to plexins with neuropilin (Nrp). However, as an exception, Sema3E binds to plexinD1 (PlxnD1) independently of Nrp. Plexin works as a GAP for the Ras and Rap family [55]. For instance, plexin activated by semaphorin inhibits R-Ras to reduce integrin activity [56]. In addition, semaphorin/plexin spatiotemporal control allows axons to pass the CNS midline without crossing back. A transient population of glutamatergic neurons around the corpus callosum produces Sema3C and provides commissural axons with an attractive force toward the contralateral hemisphere. Nrp1 is a coreceptor for VEGF-A_165_ and works as a class III semaphorin receptor [57]. Sema3A and Sema3F, which are class III semaphorins, function as antiangiogenic factors by competitively interfering with VEGFRs. In zebrafish, PlxnD1 is expressed in the vasculature during the early developmental stage. Sema3A1 and Sema3A2 are expressed in the developing somite but not the intersomatic boundaries. During angiogenesis, Sema3A/PlxnD1 negatively regulates angiogenesis via VEGF signal modulation. Endothelial cells migrate between the intersomatic boundaries [58,59]. The *out-of-bounds* (*obd^fov^*^01*b*^) mutant, which lacks *PlxnD1* expression, displays overbranched vessels because the vessels grow into the semaphorin-rich central regions of the somite [58]. This suggests that the CNS regulates repulsive signaling during angiogenesis. In semaphorin3A knockout mice, vascular branching in the cranial blood capillaries and trunk ISVs is decreased [60].

Sema3E derived from neurons restricts vascular patterning in the retina. Sema3E-PlxnD1 signaling activates small GTPase RhoJ, which sequentially retracts endothelial filopodia [61]. RhoJ is a Cdc42 subfamily member that is highly expressed in endothelial cells and induces actin depolymerization by competing with Cdc42 for their common effector proteins. GTP-RhoJ further promotes the Sema3E-induced VEGFR2-PlxnD1 association and p38 activation, which facilitates reverse cell migration.

## 7. Conclusions

Growth factors and guidance molecules regulate cell migration and morphology. When there is a lack of guidance molecules or ectopic expression, several abnormalities may arise. However, technically, it is difficult to analyze precisely because of the varieties of isoforms of VEGF and subtypes of guidance molecules and the expression patterns change dynamically. Although the interaction of ligands and receptors are revealed in in vitro assay, the actual roles in vivo are very complex. New techniques, such as imaging, genome editing, and bioinformatics, have improved our understanding of the vascular formation and neuronal formation, which has enabled more detailed classifications of endothelial cells and neurons. Furthermore, not only do endothelial cells and neurons share network formations, but they also regulate the network formations. The combination of ligands and receptors enables the maintenance of precise signaling balance.

Due to the deep wisdom regarding VEGF-A, VEGF-A is one of the major targets for therapies in patients with cancer and age-related macular degeneration (ARMD). Injection of anti-VEGF-A antibody improves eye vision in ARMD patients [62]. VEGF-A targeting monoclonal antibody Bevacizumab (Avastin^®^) is one of the first targeted therapies for solid tumors [23]. It has been anticipated that Anti-VEGF therapy is an ideal therapy for tumors. However, there are some problems. Tumor vessels are leaky and immature. Therefore, antibodies and inhibitors cannot be delivered to the target sites efficiently. Since normal endothelial cells also require VEGF-A for vessel maintenance, the inhibition of VEGF-A causes side effects. In the original hypothesis, it was anticipated that resistance would not occur. Unfortunately, resistances against anti-VEGF-A antibodies were reported, and clinical trials showed less effective results than they expected [63,64]. Combination with other therapies such as gene therapy or iPS therapy will improve the treatment.

Guidance molecules regulate not only developmental axon guidance but also axonal regeneration following injury to the nervous system. For example, EphA4 affects axonal regeneration after spinal cord injury. After spinal cord injury, EphA4 expression is upregulated, and it inhibits axonal regeneration. EphA4 blocker improves axonal regeneration in mice [65]. In addition, Slit1-Robo2 also affects neuronal regeneration. Overexpression of Slit1 in neuroblasts improves functional recovery in the poststroke mice [47]. We have to develop how to express the target gene or treat with chemicals or stem/progenitor cells in the brain. It should have the potential to improve the recovery function of patients.

Angiogenesis and neurogenesis occur during both physiological processes and pathological conditions. The identification of new factors will be valuable for future clinical research.

## Data Availability

Not applicable.
